# Clinical and radiographic evaluation of topical vitamin D application on immediate dental implants: a randomized clinical trial

**DOI:** 10.1186/s12903-025-06465-6

**Published:** 2025-07-05

**Authors:** Bassem M. Ayyad, Mohamed Said Hamed, Ahmed Elrody, Pierre A. Hanna, Ahmed Abdelmohsen Younis

**Affiliations:** 1 Oral and Maxillofacial Surgery Department, Faculty of Dentistry, Suez Canal University, Ismailia, 41522 Egypt; 2 Oral and Maxillofacial Radiology Department, Faculty of Dentistry, Suez Canal University, Ismailia, 41522 Egypt; 3 Pharmaceutics and Industrial Pharmacy Department, Faculty of Pharmacy, Suez Canal University, Ismailia, 41522 Egypt

**Keywords:** Tooth replacement, Implant surface modifications, Bioactive agents, Calcitriol emulgel, Osseointegration, Implant stability, Bone density, IDRISI kilimanjaro software

## Abstract

**Background:**

Immediate dental implants reduced treatment time and improved patient satisfaction, but achieving optimal osseointegration remained challenging. Bioactive agents, such as growth factors, cytokines and vitamins, are explored for their roles in enhancing bone healing and implant osseointegration. Vitamin D3 (calcitriol) showed promising potential in promoting these processes. This study aimed to evaluate the clinical and radiographic effects of topical vitamin D3 (calcitriol) gel on immediate dental implants.

**Methods:**

Twenty-four immediate implants were placed in healthy patients (aged 21–40 years) requiring replacement of single-rooted teeth in the anterior mandible. Patients were randomly allocated to either the control group (Group I: 12 implants inserted after tooth extraction) or the study group (Group II: 12 implants inserted after tooth extraction with topical vitamin D3 (calcitriol) gel). Clinical evaluations, including peri-implant probing depth, bleeding index, pain score and implant stability, were performed at baseline, 3 and 6 months postoperatively. Radiographic evaluation, represented by bone density analysis was conducted at the same intervals.

**Results:**

After 6 months, the Vitamin D group showed significant improvement in soft tissue healing with greater reductions in probing depth and bleeding index (1.17 mm and 0.58 vs. 1.83 mm and 1.42, respectively). Postoperative pain scores were significantly lower in the Vitamin D group by day 7 (1.00 vs. 2.50), reflecting an 81.2% reduction compared to the control group. Implant stability significantly reached 80 ISQ in the Vitamin D group compared to 75 ISQ in the control group. Bone density was significantly higher in the Vitamin D group (139.01 vs. 121.01 GSV), increasing by 18.1% vs. 6.4% in the control group.

**Conclusions:**

The reductions in peri-implant probing depth and bleeding index reflected improved soft tissue healing. The decrease in postoperative pain scores suggested potential analgesic effects. The increase in implant stability and bone density indicated enhanced osseointegration. These findings support the clinical application of topical vitamin D3 for immediate dental implant placement to promote peri-implant soft and hard tissue healing.

**Trial registration:**

NCT06806423 (registration date: January 28, 2025).

## Graphical Abstract

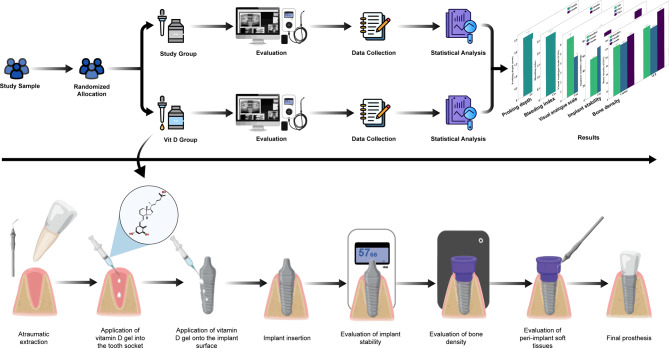

## Background

Immediate dental implant placement has gained popularity for replacing extracted teeth due to its advantages, including shorter treatment time, better preservation of alveolar bone volume and enhanced aesthetic outcomes [1]. However, the success of immediate implants largely relies on achieving optimal osseointegration, defined as the direct structural and functional bond between living bone and the surface of a load-bearing implant [2]. Factors such as implant surface properties, surgical techniques and patient-specific variables like bone quality influence this process [1, 3].

Extensive research has aimed to enhance bone-to-implant contact (BIC), a key factor for successful osseointegration. Strategies include modifying implant surface morphology and applying bioactive agents, both intended to improve BIC and support long-term implant integration [4].

Among these agents studied, vitamin D has shown significant potential as a promising candidate. Originally identified as a substance in cod liver oil that cured rickets in dogs, it exists as cholecalciferol (D3) and ergocalciferol (D2), with D3 synthesized in the skin via UV exposure or obtained from diet. It becomes biologically active after hydroxylation in the liver (to 25(OH)D) and kidneys (to 1,25(OH)_2_D or calcitriol). Calcitriol promotes osteogenesis and bone remodeling by interacting with vitamin D receptors (VDR) on osteoblasts and osteoclast precursors. This interaction regulates the production of osteogenic markers and enhances the expression of Receptor Activator of Nuclear Factor Kappa-B Ligand (RANKL), a key factor in bone resorption and remodeling [5, 6].

Calcitriol has shown promise in promoting bone regeneration. In vitro studies using a sustained-release delivery system enhanced osteogenesis, proliferation and migration of bone mesenchymal stromal cells from ovariectomized rats. In vivo, this system promoted bone regeneration in rats with femoral bone defects, showing low cytotoxicity, suitable degradation and potential for osteoporosis treatment [7]. Similarly, sustained-release vitamin D3 via poly-lactic acid (PLA) nanoparticles improved bone growth and implant integration around titanium implants in sheep, especially with bone grafts [8]. In diabetic patients, topical vitamin D3 on implants reduced crestal bone loss and slightly increased bone density, supporting better implant stability and osseointegration [9].

Mixing vitamin D with xenografts during alveolar ridge augmentation has been shown to enhance bone formation and implant stability. A study using vitamin D3 combined with xenogenic bone grafts for bony dehiscence defects reported significant increases in buccolingual ridge width and improved implant stability compared to grafts without vitamin D [10].

Despite increasing interest in vitamin D applications within implant dentistry, limited research has examined its efficacy for immediate implant placement. Most existing studies focus on animal models or in vitro experiments, leaving a gap in clinical evidence for human applications [4, 7, 8, 11]. Therefore, this study aimed to evaluate the clinical and radiographic outcomes of topical vitamin D application on immediate dental implants in healthy patients undergoing single-rooted tooth extractions in the anterior mandibular region.

## Methods

### Study design

**Fig. 1:**
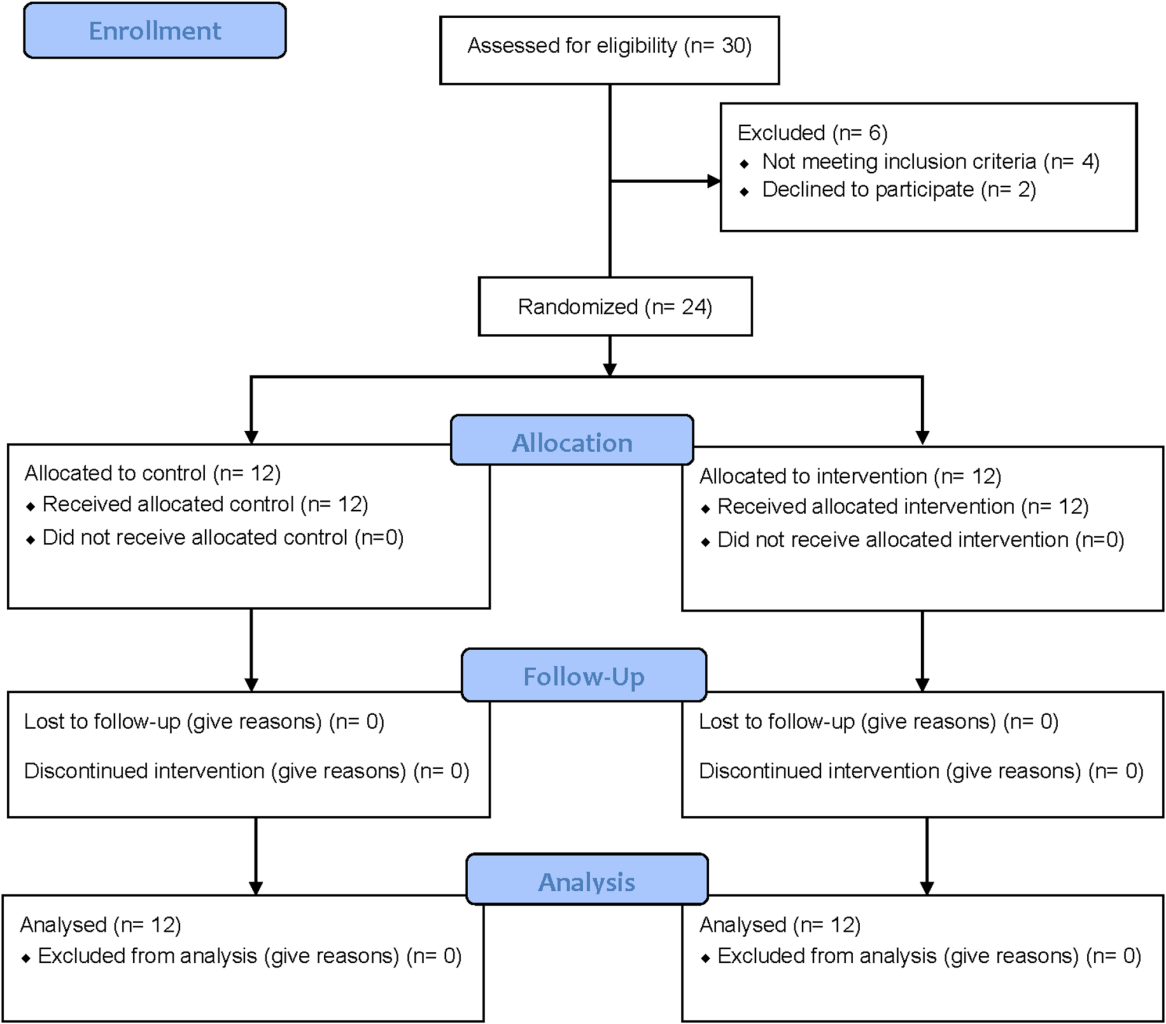
Flow diagram of the study protocol in accordance with CONSORT guidelines

This randomized clinical trial was carried out in the Oral and Maxillofacial Surgery Department at the Faculty of Dentistry, Suez Canal University, Egypt. The university’s Research Ethics Committee approved the study (Approval No. REC/534/2022). Before enrollment, all participants provided written informed consent. The research adhered fully to the principles of the Declaration of Helsinki (1975) and its 2008 revision. The trial was registered at ClinicalTrials.gov under the identification number NCT06806423 on January 28, 2025. The study was designed and reported in compliance with the CONSORT guidelines for clinical trials (Fig. 1).

### Sample size calculation

The required sample size was calculated to compare clinical and radiographic outcomes between study and control groups. Sample size analysis was conducted using G*Power software (version 3.1.9.6), with an effect size of 0.39, partial eta squared of 0.13, a power of 80% (1-β) and significance level of α = 0.05 were used as derived from previous literature [12]. The analysis indicated that a total of 24 implants (12 per group) would be sufficient to achieve the desired power, ensuring adequate sensitivity to detect meaningful differences between groups. This estimation aligns with the methodology adopted in similar studies [13].

### Patient selection

Patients requiring extraction of non-restorable single-rooted teeth in the mandibular anterior region were recruited for the study. Participants were chosen according to specific eligibility criteria, requiring them to be healthy individuals classified as ASA I by the American Society of Anesthesiologists, aged 21–40 years, with teeth or roots indicated for extraction in the specified region. Patients were selected with a thick soft tissue phenotype and a minimum keratinized tissue width (KTW) of 2 mm, assessed clinically during preoperative examination, to ensure predictable healing and favorable outcomes. These criteria ensured a consistent and relevant study population for evaluating the clinical and radiographic outcomes of topical vitamin D application on immediate dental implants.

Exclusion criteria eliminated individuals with factors that could compromise study outcomes, including pregnant or lactating females, poor oral hygiene, severe periodontal disease, patients with bone or soft tissue defects, or harmful oral habits such as heavy smoking or bruxism. Patients with infections or pathological changes in teeth targeted for immediate implant placement were also excluded to ensure the safety and reliability of the study.

### Study groups and randomization

The study included 24 immediate implants replacing non-restorable single-rooted teeth in the anterior mandibular region. The patients were randomly assigned in equal numbers to two groups using www.randomizer.org:

**Group I (Control): 12** implants were inserted immediately after teeth extraction.

**Group II (Study): 12** implants were inserted immediately after teeth extraction, combined with the application of topical vitamin D3 (calcitriol) gel.

### Preoperative phase

#### Clinical examination

Personal data, including name, age, gender, occupation and contact information, were collected for each patient. A detailed medical and dental history was taken to exclude those with conditions or habits that could negatively impact implant stability.

Oral hygiene was assessed and patients were referred to the Oral Medicine and Periodontology Department for scaling and polishing. They underwent professional oral prophylaxis and were instructed to maintain optimal oral hygiene throughout the study. Occlusion was evaluated to ensure proper alignment and contact. The width of the alveolar ridge was also assessed clinically.

The surgical procedure and potential complications (e.g., bleeding, pain, swelling, implant failure) were explained to each patient. Photographs and follow-up X-rays were used for documentation and monitoring healing. These data were collected to ensure each patient met the inclusion criteria.

#### Radiographic examination

Preoperative direct digital panoramic radiographs were used for initial screening to assess bone quality and alveolar ridge height. Patients with severe bone loss or insufficient alveolar height were excluded. Bucco-lingual width was evaluated clinically during the surgical phase, using a periodontal probe to measure the width of the extraction socket and residual bone.

### Preparation of calcitriol gel

First, disodium hydrogen phosphate (3.11 g), citric acid (0.137 g), cremophor RH 40 (0.75 g) and tween 80 (0.35 g) were dissolved in 100 mL of distilled deionized water. Cellosize™ K100M (0.75 g) and PVP K90 (1.5 g) were then dispersed to the solution while stirring at 300 rpm for 4 h. Glycerol (3.5 g) was then added as viscosity enhancer. The gel formulation was kept overnight under stirring at 200 rpm. The appropriate amount (8 mL) of the drug’s oily solution (1 µg/mL) was then added. Ultimately, deionised distilled water was used to raise the final gel formulation’s volume to the appropriate level [10].

The produced emulgels were sterilised using a combination of aseptic mixing and heat sterilisation. Saturated water steam was used to sterilise the plain gel base and the calcitriol oily solution, separately. The two phases were then left to cool until reaching room temperature followed by aseptic mixing in a sterile booth with laminar airflow. Sterile gels were then packed and kept in sterile syringes and kept in a refrigerator at 4 °C.

The high-Performance Liquid Chromatography (HPLC) method described by Temova and Roškar was used to detect calcitriol concentrations [14]. A mobile phase consisting of acetonitrile and methanol (90:10, v/v) was passed through a reversed-phase Gemini C18 column (100 × 3.0 mm, 3 μm particle size, Phenomenex, USA) at a flow rate of 1 mL/min. Detection was done at 265 nm (Agilent Technologies, Series 1100/1200, USA). Each gel base was visually examined against a dark, backlit background to evaluate its relative transparency, the presence of emulsified oil globules and the overall homogeneity of its structure, serving as a standard method for gel base quality control.

The pH of the prepared solution was measured at room temperature using a calibrated pH meter (827 PH Lab, Metrohm, Switzerland) after diluting 1 g of each gel in 100 mL of double-distilled water. This was done to ensure that the gel’s pH was compatible with the physiological pH, which is defined as being in the range of 7.2–7.4 [15].

The gel viscosity was assessed using an AMETEK Brookfield DV3TRVCP, USA, rheometer [16]. The spindle was inserted into the sample and rotated at a speed of 1 RPM while the sample holder was filled with gel. At RT, rheological analyses were conducted (*n* = 3). With some minor adjustments, the analysis of the produced gels’ spreadability potential was carried out using data from a published work [17].

Using this technique, 2 g of gel was put in the middle of two lines spaced 4 cm apart on a regular glass slide. Next, a second, 110-grame glass slide was delicately set on top of the gel. From the time the second glass slide was placed until the gel spread evenly between the two lines, the dispersion time was computed. The experiment was conducted in three iterations and the interim results were calculated.

The spreadability was determined using the following formula (Eq. 1):

$$\:S=W\:\times\:\:\frac{d}{t}$$

where S represents spreadability, W is the weight of the upper slide (g), d is the distance between the two lines (cm) and t is the time (seconds).

The test was as simple as pouring the mixtures into collapsable metal tubes. Next, the tubes were compressed to extrude a 0.5-cm gel ribbon and the hydrogels’ extrudability time was recoded [18].

Deionised distilled water was prepared up to 100 mL and used to carefully weigh 1 g of gel formulation in order to measure the medication drug content (DC). After filtration through a 0.45 μm filter and dilution to a ratio of 1:5, the drug’s concentration was ascertained by means of the HPLC technique [19].

(DC) percentage was determined using the following formula (Eq. 2):

$$\>DC\left( \% \right) = \>{{\left( \matrix{{\rm{Detected}}\>{\rm{drug}}\>{\rm{content}}\> \hfill \cr {\rm{in}}\>{\rm{the}}\>{\rm{gel}} \hfill \cr} \right)} \over {\left( \matrix{{\rm{Initial}}\>{\rm{drug}}\>{\rm{content}}\> \hfill \cr {\rm{added}}\>{\rm{to}}\>{\rm{the}}\>{\rm{gel}} \hfill \cr} \right)}}\> \times \>100\>$$

Seventy-two hours after the gel formulation was prepared, samples were taken from five different spots on the drug-loaded gel to ensure uniformity of content. The quantification method was then used to determine the drug’s amount.

The in vitro release of calcitriol from the formulated gel was analyzed using the shaking bottle method. A precisely measured portion of the gel was put into the dialysis bag, which was then sealed tightly at both ends and put inside a glass vessel. The glass containers holding 100 millilitres of pH 6.8 phosphate buffet solution with 1% tween 80 were heated in a shaking water bath while the dissolution media were being prepared. A temperature adjustment was made to 37 ± 0.5 °C in the water bath. About 100 rpm was chosen as the water bath’s shaking speed [20].

Using a pipette, samples of the dissolving media were taken out of each jar at a prearranged time. The sample that was removed weighed one millilitre. Each vial’s lost volume was replenished with an identical volume of a brand-new dissolving liquid that had been warmed to the same temperature using the same water bath. The previously mentioned HPLC procedure was then used to arrange the samples for analysis.

### Surgical phase

**Fig. 2:**
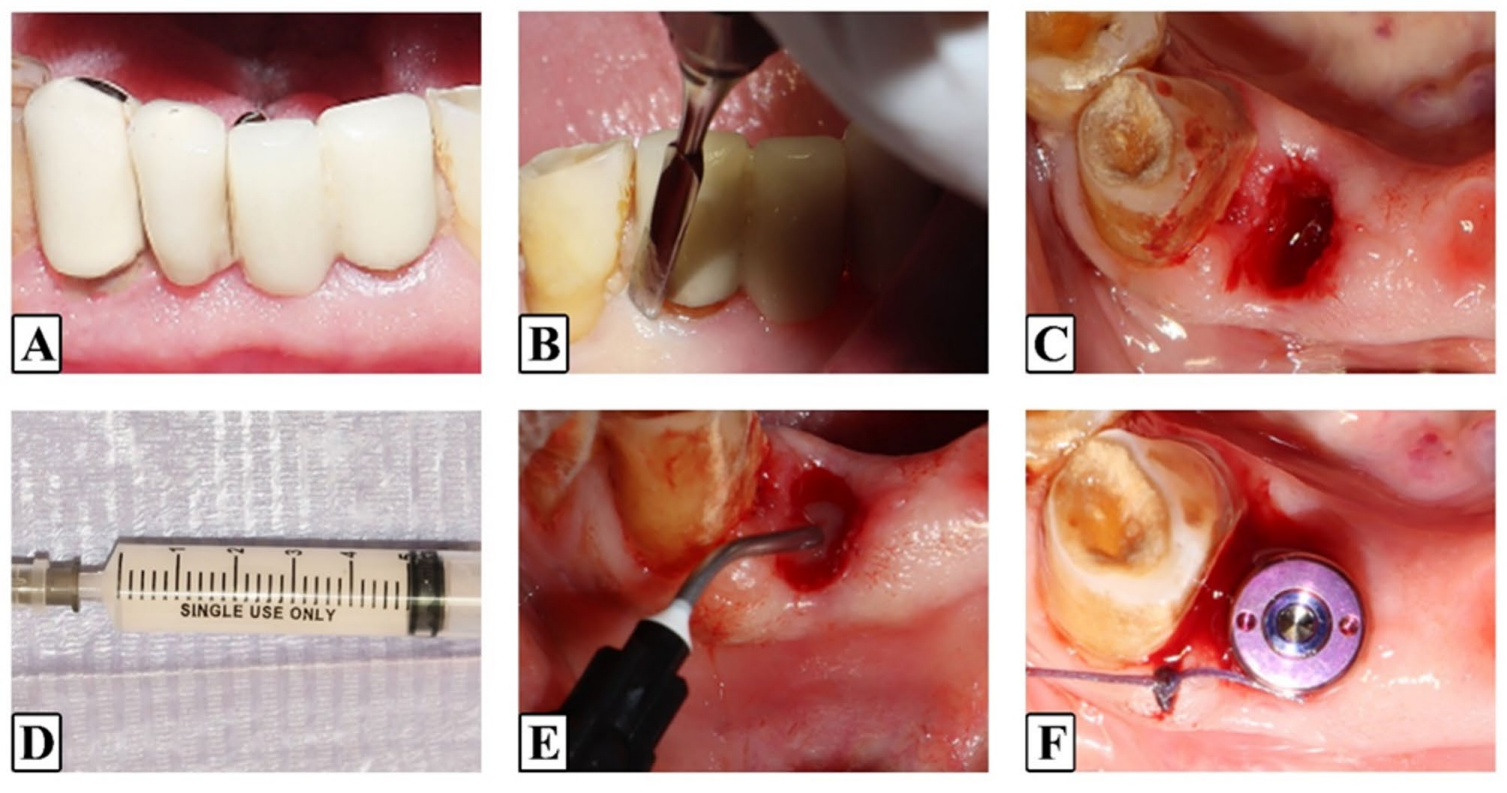
Photographs of the surgical procedure **A.** Pre-operative Group II **B**. Atraumatic extraction with periotome **C**. Preserved tooth socket **D.** Sterile Vitamin D gel syringe **E.** Application of Vitamin D gel **F.** Healing abutment and suturing

The surgical procedures, as shown in Fig. 2, were carried out following strict infection control protocols. Patients were instructed to rinse with 0.1% chlorhexidine (Hexitol antiseptic mouthwash, Arabian Drug Company (ADCO), Cairo, Egypt) and the peri-oral tissues were disinfected with povidone-iodine (Betadine, Nile Co. for Pharma, Cairo, Egypt). Local anesthesia was injected via the infiltration technique using 4% articaine hydrochloride with 1:100,000 epinephrine (Artinibsa, Inibsa Dental SLU, Spain).

Atraumatic tooth extractions were conducted with periotomes and anterior forceps, preserving the alveolar bone. The dimensions of the extraction socket and residual bone were evaluated using a periodontal probe, in addition to measuring the width and length of the extracted tooth root, to ensure appropriate implant selection. Measurements were taken to confirm compatibility with the planned implant size and ensure proper placement.

Osteotomy drilling followed a standardized protocol, using sequential drills at 950–1100 rpm with 35 Ncm torque under copious irrigation.

In the study group, sterile vitamin D3 (calcitriol) gel was applied to both the implant surface and the osteotomy site immediately before placing the implant. ROOTT R-line implants (TRATE AG, Switzerland) were chosen based on socket size and required primary stability. The implants were carefully inserted into fresh extraction sockets and their alignment was verified to ensure correct positioning. In both the study and control groups, bone grafting was not performed at the jumping gap to isolate the effects of vitamin D.

Implant stability was assessed using the Osstell device (Osstell AB, Gothenburg, Sweden) to measure implant stability quotient (ISQ) values, which were measured immediately after implant insertion as baseline readings. Large healing abutments were immediately placed on the implants and secured with simple interrupted 3 − 0 Vicryl sutures (EGYSORB, Egypt) to ensure complete closure of the surgical site, minimizing the risk of leakage and preserving the soft tissue emergence profile without tension.

### Postoperative phase

#### Post-surgical instructions

Following surgery, patients received detailed postoperative care instructions and prescribed medications including Cataflam 50 mg tablets (Diclofenac potassium) a non-steroidal anti-inflammatory drug taken twice daily for 4 days and Hexitol mouth wash (Chlorhexidine HCL 0.12%) an antiseptic mouth wash used twice daily starting the second day after surgery for 7 days.

#### Post-operative evaluation

**Fig. 3:**
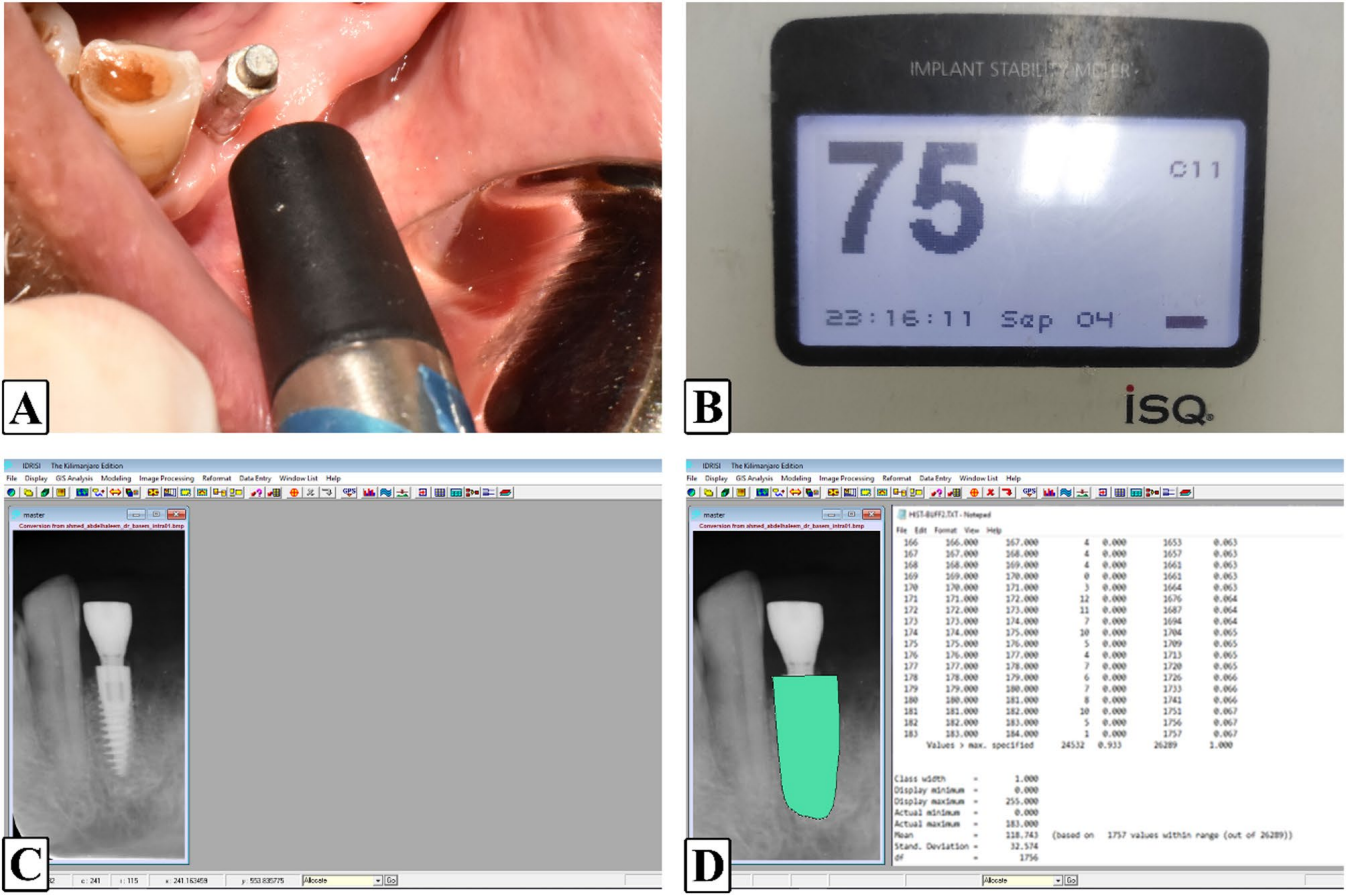
Photographs of post-operative evaluation **A.** Implant stability assessment with Osstell ISQ device and smart peg **B.** Osstell ISQ system readings **C.** Bone density assessment with a digital periapical radiograph using IDRISI Kilimanjaro software **D.** Bone density values

The examiners performing clinical and radiographic assessments were blinded to the group assignments. Follow-up appointments were scheduled throughout the first week, as well as at 3 months and 6 months postoperatively. During each visit, the following parameters were evaluated (Fig. 3):

1. Clinical Assessments:

Probing depth and bleeding index measurements were recorded at 3 and 6 months to assess peri-implant soft tissue healing. Pain levels were assessed using a Visual Analog Scale (VAS) on the 1st, 3rd and 7th days postoperatively. Implant stability was reassessed at 3 and 6 months using the Osstell ISQ device.

2. Radiographic Assessment:

Standardized digital periapical radiographs were obtained using a Rinn XCP film holder with parallel technique to ensure consistent angulation and image quality at baseline, 3 months and 6 months to evaluate bone density changes around the implants. The IDRISI Kilimanjaro software was used to analyze grayscale values, providing quantitative measurements of bone density.

### Statistical analysis

The data were analyzed and visualized using SAS JMP statistical software version 17.2. Descriptive statistics are presented as mean ± standard deviation. To compare outcomes between the vitamin D and control groups at each individual time point, independent Student’s T-tests were performed. Each time point was analyzed separately, in line with the study’s objective of evaluating between-group differences at distinct intervals. A confidence interval of 95% was used in the statistical analysis leading to p-value below 0.05 was considered statistically significant.

## Results

### Patient demographics distribution

The study involved healthy patients classified as ASA I, with an average age of 35 ± 1.69 years. A total of 24 implants were evenly distributed between the control group (Group I) and the study group (Group II), ensuring balanced demographics as shown in Table 1. The mean age in the control group was 35.17 ± 2.04 years and in the vitamin D group was 34.83 ± 1.34 years. The difference was not statistically significant (*p* = 0.64). All participants successfully completed the six-month follow-up without implant failures or major complications.

**Table 1:** Demographic data and sample distribution (*n* = 24)

Case	Group	Age	Tooth
1	Control	36	41
2	Vit D	35	32
3	Control	36	33
4	Vit D	37	42
5	Control	35	31
6	Vit D	35	43
7	Control	40	42
8	Vit D	35	33
9	Control	35	32
10	Vit D	36	41
11	Control	34	43
12	Vit D	34	31
13	Control	35	41
14	Vit D	33	32
15	Control	33	33
16	Vit D	34	42
17	Control	34	31
18	Vit D	37	43
19	Control	32	32
20	Vit D	34	33
21	Control	37	41
22	Vit D	35	31
23	Control	35	42
24	Vit D	33	41

### Clinical outcomes

#### Soft tissue healing

##### Peri-implant probing depth

Table 2 presents the comparison of peri-implant probing depths (PD) between the control and Vitamin D groups at 3 and 6 months postoperatively. At 3 months, the control group showed a mean probing depth of 2.42 ± 0.51 mm, while the Vitamin D group had a slightly lower mean of 2.00 ± 0.60 mm. By 6 months, both groups demonstrated a reduction in probing depth, with the control group averaging 1.83 ± 0.72 mm and the Vitamin D group showing a more pronounced decrease to 1.17 ± 0.72 mm. This represents a 41.5% reduction in probing depth for the Vitamin D group compared to 24.4% in the control group. A significant statistical difference was noted at 6 months (*p* = 0.033), suggesting improved peri-implant soft tissue healing in the Vitamin D group.

**Table 2:** Comparison of probing depth between control and study groups at 3 and 6 months (mean ± SD, *n* = 24)

Group	Probing depth (mm)	
	3 months	6 months
Control	2.42 ± 0.51	1.83 ± 0.72
Vit D	2.00 ± 0.60	1.17 ± 0.72
t Ratio	-1.8202	-2.2752
P-Value	0.0827	0.033*

##### Bleeding index

The bleeding index (BI) values for both groups at 3 and 6 months are shown in Table 3. At 3 months, the control group had a mean bleeding index of 2.50 ± 0.52, compared to 1.75 ± 0.45 in the Vitamin D group. By 6 months, the bleeding index further decreased in both groups, with values of 1.42 ± 0.51 in the control group and 0.58 ± 0.51 in the Vitamin D group. The Vitamin D group exhibited a 66.9% reduction compared to 43.2% in the control group. The groups exhibited statistically significant differences at both time points (*p* = 0.0011 at 3 months, *p* = 0.0007 at 6 months), indicating a greater reduction in inflammation for the Vitamin D group.

**Table 3:** Comparison of bleeding index between control and study groups (mean ± SD, *n* = 24)

Group	Bleeding index	
	3 months	6 months
Control	2.50 ± 0.52	1.42 ± 0.51
Vit D	1.75 ± 0.45	0.58 ± 0.51
t Ratio	-3.7607	-3.9641
P-Value	0.0011*	0.0007*

#### Postoperative pain

Table 4 summarizes postoperative pain levels, measured using the Visual Analogue Scale (VAS), at 1, 3 and 7 days post-surgery. On the first day, the control group reported a mean pain score of 6.17 ± 1.47, whereas the Vitamin D group had a slightly lower mean of 5.33 ± 0.98. By the third day, pain levels reduced in both groups, but a significant difference emerged, with scores of 3.83 ± 0.94 in the control group and 2.58 ± 0.67 in the Vitamin D group (*p* = 0.0012), showing a 51.6% pain reduction in the Vitamin D group compared to 37.9% in the control group. By the seventh day, pain levels had significantly declined, with the control group at 2.50 ± 0.67 and the Vitamin D group at 1.00 ± 0.74 (*p* < 0.0001), indicating an overall reduction of 81.2% in the Vitamin D group versus 59.5% in the control group, based on VAS scores.

**Table 4:** VAS scores at 1st, 3rd and 7th days post-surgery (mean ± SD, *n* = 24)

Group	Visual Analogue Scale (VAS)		
	1 day	3 days	7 days
Control	6.17 ± 1.47	3.83 ± 0.94	2.50 ± 0.67
Vit D	5.33 ± 0.98	2.58 ± 0.67	1.00 ± 0.74
t Ratio	-1.634	-3.7607	-5.1962
P-Value	0.1185	0.0012*	< 0.0001*

#### Implant stability

Table 5 provides detailed data on implant stability measurements. The Osstell device was used to assess implant stability, presented as implant stability quotient (ISQ) values. During implant placement, these values were comparable between groups (50.00 ± 5.59 in the control group vs. 53.92 ± 5.93 in the Vitamin D group, *p* = 0.1103). By 3 months, the Vitamin D group demonstrated a significantly higher stability score (64.25 ± 3.39) compared to the control group (60.58 ± 2.27, *p* = 0.0057). This trend continued at 6 months, with the Vitamin D group reaching an average stability of 80.00 ± 3.86, significantly greater than the control group’s 75.00 ± 2.13 (*p* = 0.0011).

**Table 5:** Osstell readings for both groups at immediate assessment, after 3 months and 6 months (mean ± SD, *n* = 24)

Group	Implant stability (ISQ)		
	Immediate	3 months	6 months
Control	50.00 ± 5.59	60.58 ± 2.27	75.00 ± 2.13
Vit D	53.92 ± 5.93	64.25 ± 3.39	80.00 ± 3.86
t Ratio	1.664446	3.112685	3.926902
P-Value	0.1103	0.0057*	0.0011*

### Radiographic outcome

#### Bone density

Bone density measurements at immediate placement, 3 months and 6 months are shown in Table 6. Gray Scale Value (GSV) is used as a radiographic measure of bone density, representing brightness intensity in grayscale images, with higher values indicating denser bone. Initial bone density values were similar between the control (114.30 ± 16.75) and Vitamin D groups (118.06 ± 13.73, *p* = 0.5532). At 3 months, a slight reduction was noted in both groups, with no significant difference (*p* = 0.653). However, by 6 months, the Vitamin D group exhibited a significant increase in bone density (139.01 ± 13.76) compared to the control group (121.01 ± 13.03), reaching statistical significance (*p* = 0.0257). The increase in bone density suggests that topical Vitamin D enhanced bone mineralization by supporting calcium deposition and osteoblast activity, leading to improved osseointegration and bone regeneration.

**Table 6:** Bone density measurements at immediate, 3 and 6 months (mean ± SD, *n* = 24)

Group	Bone density (GSV)		
	Immediate	3 months	6 months
Control	114.30 ± 16.75	111.24 ± 14.07	121.01 ± 13.03
Vit D	118.06 ± 13.73	108.97 ± 9.87	139.01 ± 13.76
t Ratio	0.602623	-0.45659	0.0034
P-Value	0.5532	0.653	0.0257*

#### Percentage of change

Table 7 displays the percentage change in bone density over time. In the first quarter (0–3 months), both groups experienced a decrease in bone density, with the Vitamin D group showing a greater reduction (-7.27 ± 6.54%) compared to the control group (-2.33 ± 5.28%, *p* = 0.0543). However, during the second quarter (3–6 months), the Vitamin D group demonstrated a significantly greater increase (27.60 ± 5.69%) relative to the control group (9.26 ± 8.00%, *p* < 0.0001). Overall (0–6 months), the Vitamin D group exhibited a significantly higher net increase in bone density (18.07 ± 5.65%) compared to the control group (6.44 ± 5.12%, *p* < 0.0001), reinforcing the positive impact of Vitamin D on bone healing and regeneration.

**Table 7:** Percentage changes in bone density over time (mean ± SD, *n* = 24)

Group	Percentage of change (%)		
	1st quarter	2nd quarter	Overall
Control	-2.33 ± 5.28	9.26 ± 8.00	6.44 ± 5.12
Vit D	-7.27 ± 6.54	27.60 ± 5.69	18.07 ± 5.65
t Ratio	-2.03784	6.475508	5.287272
P-Value	0.0543	< 0.0001*	< 0.0001*

### Characterization and evaluation of calcitriol emulgel

The calcitriol-loaded emulgel exhibited a white colour, while the plain gel was clear and transparent. The formulation showed no visible greasy globules, indicating a homogeneous distribution with extremely small particle size. The pH of the emulgel was measured at 7.28 ± 0.10, which is consistent with the physiological pH range of 7.2 to 7.4, ensuring compatibility with the human body.

The viscosity of the emulgel was recorded as 3912.7 ± 234.79 cps, which is suitable for topical applications, allowing ease of handling without impeding drug release. The spreadability was measured at 15.43 ± 0.85 g.cm/sec, indicating a consistent gel formulation that spreads smoothly across surfaces. Extrudability tests demonstrated that the gel could be easily released from its packaging, with 0.5 cm of gel being extruded within 15 s under light pressure.

**Fig. 4:**
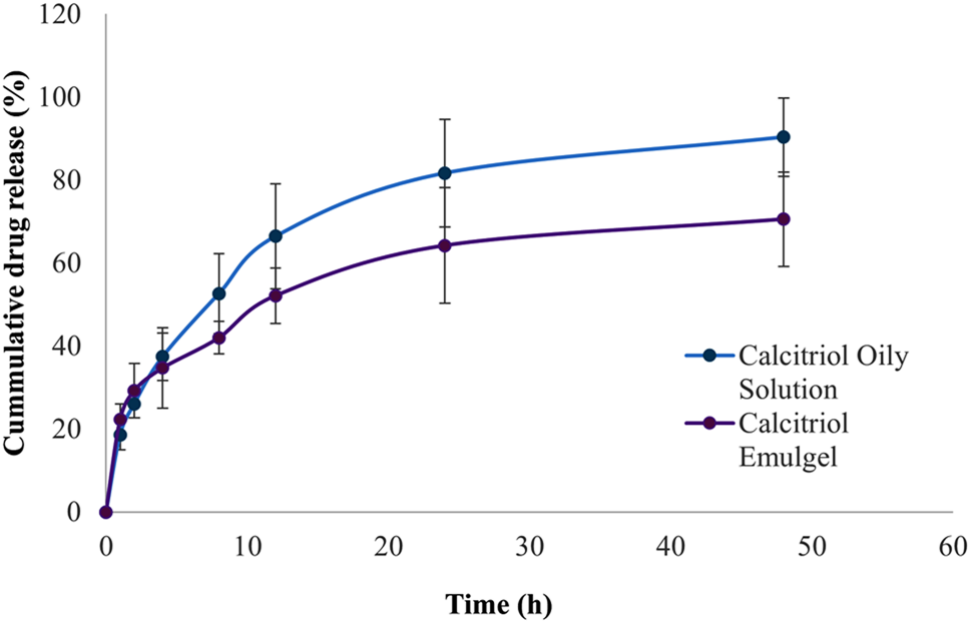
In vitro drug release profile form emulgel vs. standard reference oily solution

The amount of calcitriol in the emulgel was measured accurately following the construction of a calibration curve using HPLC, ensuring reliable quantification of the drug. The drug content ranged between 95.47% and 101.54% of the theoretical value, confirming uniform distribution and phase consistency within the gel. The in vitro drug release studies exhibited a rapid initial release of calcitriol, followed by a sustained release (Fig. 4). This biphasic release pattern ensures a high concentration of the drug in the initial phase, promoting rapid osteogenesis, while the slow release ensures prolonged availability of the drug for continuous treatment. The physicochemical properties of the calcitriol-loaded emulgel are summarized in Table 8.

**Table 8:** Physicochemical properties of the calcitriol-loaded emulgel

Parameter	Value
pH	7.28 ± 0.10
Viscosity	3912.7 ± 234.79 (cps)
Spreadability	15.43 ± 0.85 g.cm sec^− 1^
Extrudability	16.47 ± 1.66 s
Drug content	97.94 ± 3.19%

## Discussion

Immediate implant placement following tooth extraction has gained significant attention due to its ability to preserve alveolar bone, prevent soft tissue collapse and reduce treatment time. This approach is important for maintaining the integrity of the extraction socket and eliminating the need for future bone augmentation procedures, which directly influence both functional and aesthetic outcomes [21].

Bioactive agents, including growth factors, morphogenetic proteins and vitamins, have been extensively studied for their potential in enhancing peri-implant bone formation. Vitamin D is particularly significant in bone metabolism and regeneration by regulating osteoblast differentiation and function, promoting bone formation and modulating osteoclast activity to facilitate bone remodeling. This balanced regulation is essential for maintaining bone homeostasis and improving implant osseointegration [22, 23].

In this study, 24 implants were placed across patients classified as ASA I to evaluate the clinical and radiographic effects of topical vitamin D3 (calcitriol) gel on immediate dental implants, ensuring consistency in surgical protocols and postoperative care. This sample size aligns with Erdem et al. (2023), who reported favorable implant stability and bone formation outcomes [24]. Similarly, Kim et al. (2022) assessed the accuracy of digital surgical guides using 24 implants, reinforcing the adequacy of this sample size for evaluating implant-related parameters [13].

Middle-aged patients were selected for their relatively stable bone turnover rates and minimal hormonal changes, allowing a more precise assessment of vitamin D’s effects on implant osseointegration. Liapaki et al. (2022) similarly found that middle-aged individuals exhibit more predictable implant success rates compared to older populations, supporting this study’s methodology [25].

Focusing on the anterior mandible provided distinct advantages: uniform bone density, consistent occlusal forces, comparable socket dimensions and uniformity in bone quality and surgical conditions. These factors minimized anatomical variability, standardized procedures and improved the reliability of implant performance assessment. The region’s high bone density supported superior primary stability and predictable osseointegration while enabling precise localized application of topical vitamin D gel with minimal unintended spread. This targeted approach yielded robust reproducible outcomes. Wang et al. (2023) confirmed that denser anterior mandibular bone significantly enhances primary implant stability. Although this focus may limit generalizability to regions like the posterior mandible or maxilla with varying bone characteristics, it strategically prioritized precision and internal validity, establishing a reliable model for implant success that guides future research across diverse anatomical sites [26].

The inclusion and exclusion criteria ensured a homogeneous patient population, free from systemic diseases or compromised oral hygiene that could confound results. Patients with severe periodontal diseases, periapical infections, smoking habits, or bruxism were excluded to minimize negative impacts on implant soft and hard tissues healing outcomes. These criteria align with Alterman et al. (2023), who reviewed risk factors for early implant failure [27].

The topical calcitriol emulgel formulated in this study was based on Amr (2019) to ensure stability and effective local delivery. The emulgel exhibited a viscosity that balanced spreadability and sustained drug release, with a pH ensuring biocompatibility. The in vitro release profile demonstrated an initial rapid release followed by sustained release, likely due to the combination of surfactants and gelling agents used. This formulation was designed to enhance bone regeneration and implant osseointegration [10].

Resonance frequency analysis (RFA) using the Osstell device at baseline, three months and six months postoperatively provided reliable, non-invasive quantifications of implant stability measurements. This method aligns with studies by Bavetta et al. (2024), which validated ISQ values as reliable indicators of immediate implant success [28].

Direct digital panoramic radiographs were used for initial screening to assess anatomical landmarks and patient eligibility in the anterior mandibular area. This imaging modality offers adequate diagnostic accuracy while reducing radiation exposure, improving patient safety, and lowering costs. CBCT was avoided to minimize radiation exposure and cost, following the ALARA (As Low As Reasonably Achievable) principle, despite the slight reduction in diagnostic precision that can be compensated during the surgical phase. Coşkun & Topbaş (2023) emphasized the clinical success of panoramic radiographs for preoperative dental implant assessment, supporting their reliability for initial screening [29].

Standardized digital periapical radiographs were used to assess peri-implant bone density at baseline, three months and six months post-operatively. This imaging modality offers several advantages, including adequate diagnostic accuracy, reduced radiation exposure, improved patient safety and cost-effectiveness. Lubis et al. (2023) highlighted the reliability of digital periapical radiographs for monitoring localized peri-implant bone density changes, supporting the methodology of the present study [30].

Bone density measurements were quantitatively analyzed using Idrisi Kilimanjaro software, known for its precision in radiographic images analysis. Harhash & Tarek (2019) highlighted this software’s efficacy in measuring osseointegration and bone healing, supporting its application in this study [31].

Peri-implant soft tissue healing was assessed using probing depth (PD) alongside bleeding index (BI) as clinical indicators of inflammation and soft tissue response. These metrics are well-established in clinical practice for evaluating gingival health and inflammation around implants. Heeba et al. (2019) demonstrated the reliability of PD and BI metrics in monitoring peri-implant health, particularly in controlled diabetic patients [9].

The results of this study showed enhanced soft tissue healing in the topical vitamin D-treated group, which exhibited significantly lower PD and BI values at three and six months postoperatively, suggesting improved soft tissue healing and a reduced risk of peri-implantitis. These findings align with Heeba et al. (2019), who also observed enhanced peri-implant soft tissue health following vitamin D application. The observed improvements may be attributed to vitamin D’s anti-inflammatory effects and its role in collagen synthesis, which collectively enhance peri-implant tissue integration [9].

Postoperative pain was assessed using the Visual Analog Scale (VAS) on the first, third and seventh days post-surgery. The vitamin D-treated group reported significantly lower pain scores, with an 81.2% reduction by day seven compared to 59.5% in the control group. The observed pain reduction may be attributed to vitamin D’s ability to modulate inflammatory responses, potentially contributing to reduced pain perception. These findings are consistent with Al-Attar & Abid (2022), who reported significant pain relief following vitamin D3 application in dental surgeries [32].

Implant stability, assessed using Osstell’s Implant Stability Quotient (ISQ), demonstrated greater improvements in the vitamin D group. By six months, ISQ values reached 80.00 ± 3.86 in the vitamin D group, significantly higher than 75.00 ± 2.13 in the control group (*p* = 0.0011). This aligns with findings from Amr (2019), who reported enhanced implant stability following topical vitamin D3 application. While Amr’s study incorporated bone grafts mixed with vitamin D, the present findings highlight the potential of vitamin D in promoting early osseointegration [10].

Bone density analysis showed an overall increase of 18.1% in the vitamin D group, whereas the control group exhibited a 6.4% increase after six months. These findings align with Heeba et al. (2019), who observed significant improvements in bone density following vitamin D application. Similarly, Amr (2019) reported increased bone quality and buccolingual ridge width in vitamin D-treated patients, although the use of bone grafts in Amr’s study likely contributed to the more pronounced improvements [9, 10].

Further supporting evidence comes from Salomó-Coll et al. (2018), who demonstrated a 9% increase in bone-to-implant contact (BIC) with a 10% vitamin D solution applied to implant surfaces in Foxhound dogs. While some variations exist across studies, differences in vitamin D formulations and delivery methods may explain these discrepancies [4].

In contrast, Bozoğlan & Dundar (2021) found no statistically significant differences in BIC ratios in their experimental study on rats, highlighting the variability of vitamin D efficacy depending on study models and conditions, which may be due to species-related differences in bone physiology [11].

This study has some limitations that should be acknowledged. Focusing implant placement at the anterior mandible may reduce generalizability to other regions. Using CBCT could have allowed a broader bone evaluation. Parameters such as marginal bone loss and keratinized mucosa width could provide further insight into peri-implant tissue health and esthetic outcomes. These aspects may be valuable to explore in future research to enhance clinical relevance and applicability.

## Conclusions

The significant reductions in peri-implant probing depth and bleeding index suggested improved peri-implant soft tissue healing. The notable decrease in postoperative pain levels indicated the potential analgesic effects of vitamin D3, contributing to enhanced patient comfort and recovery. Furthermore, the significant improvements in implant stability and bone density highlighted its role in promoting osseointegration. These findings demonstrated that topical application of vitamin D3 enhanced peri-implant tissue healing, implant stability and bone density throughout the study period, supporting its inclusion in clinical protocols for implant dentistry, especially in cases requiring immediate placement, aims to enhance soft and hard tissue healing for optimal outcomes.

## Data Availability

All data obtained and examined throughout this study are analyzed and provided within this manuscript.
